# Origem Anômala da Artéria Coronária Circunflexa da Artéria Pulmonar Direita: Diagnóstico por TC Cardíaca

**DOI:** 10.36660/abc.20200060

**Published:** 2021-07-14

**Authors:** Bebiana Faria, Lucy Calvo, Sílvia Ribeiro, Catarina Ruivo, António Lourenço

**Affiliations:** 1 Hospital da Senhora da Oliveira Guimarães Departamento de Cardiologia Guimarães Portugal Departamento de Cardiologia, Hospital da Senhora da Oliveira Guimarães, Guimarães - Portugal; 2 Leiria Hospital Center Departamento de Cardiologia Leiria Portugal Departamento de Cardiologia, Leiria Hospital Center, Leiria - Portugal

**Keywords:** Doença da Artéria Coronariana, Anormalidades Congênitas, Cardiopatias Congênitas, Vasos Coronários, Artéria Pulmonar, Tomografia Computadorizada/métodos

## Introdução

A origem anômala da artéria coronária circunflexa esquerda (CXE) da artéria pulmonar (AP) direita é uma anomalia coronária extremamente rara. Embora o curso clínico possa ser silencioso, o risco de morte cardíaca súbita está aumentado. Os sintomas estão relacionados à colateralização e à quantidade do miocárdio que ela fornece, sendo que alguns pacientes necessitam de tratamento cirúrgico.^1^^,^^2^

## Relato de Caso

Uma mulher de 44 anos com histórico familiar de síndrome de Brugada e nenhum outra histórico médico relevante foi examinada no nosso hospital devido a angina de esforço classe I da *Canadian Cardiovascular Society*. Ela não apresentava fatores de risco coronariano e não havia histórico familiar de doença arterial coronariana prematura ou doença cardíaca congênita.^3^ O exame físico estava normal. A ecocardiografia transtorácica revelou apenas regurgitação mitral leve e o ECG estava normal.

A paciente foi submetida à ecocardiograma de esforço que revelou anormalidades de movimentação da parede ínfero-lateral, com piora do grau de regurgitação mitral. Foi excluída síndrome de Brugada após teste farmacológico com ajmalina.

Foi realizada angiotomografia de artérias coronárias (ATAC) (*Siemens Somatom Sensation 64 CT Scanner®*). Foi realizada uma varredura preliminar para marcar a quantidade de cálcio coronário e a pontuação de Agatston foi zero. Foram administrados 70 mililitros de contraste iodado (*Ultravist 370®*), bem como 0,3 mg de nitroglicerina por via sublingual imediatamente antes da injeção de contraste. A paciente estava em ritmo sinusal com frequência cardíaca de 50 a 60 batimentos/minuto. Realizou-se ATAC controlada retrospectiva, com reconstrução das fases cardíacas a 70% do intervalo R-R. O pós-processamento da imagem foi realizado no *Aquarius Intuition TeraRecon®*.

**Figura 1:**
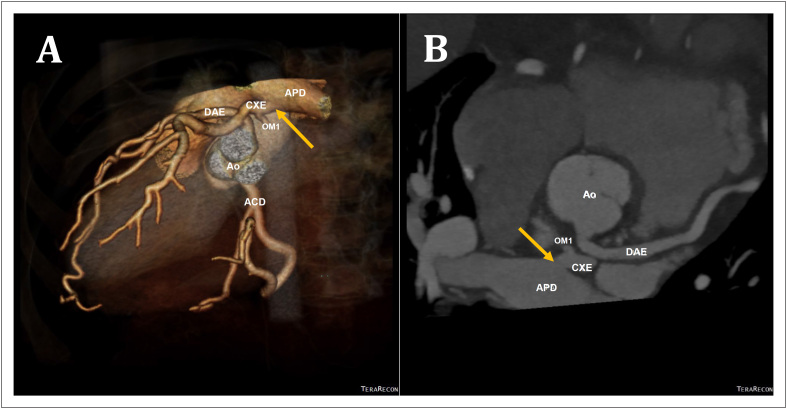
TC cardíaca em múltiplos cortes usando varredura de 64 cortes, mostrando a origem anômala da artéria coronária circunflexa da artéria pulmonar direita (flecha). ACD: artéria coronária direita; Ao: aorta; APD: artéria pulmonar direita; CXE: artéria coronária circunflexa esquerda; DAE: artéria descendente anterior esquerda; OM1: primeiro marginal obtuso.

**Figura 2:**
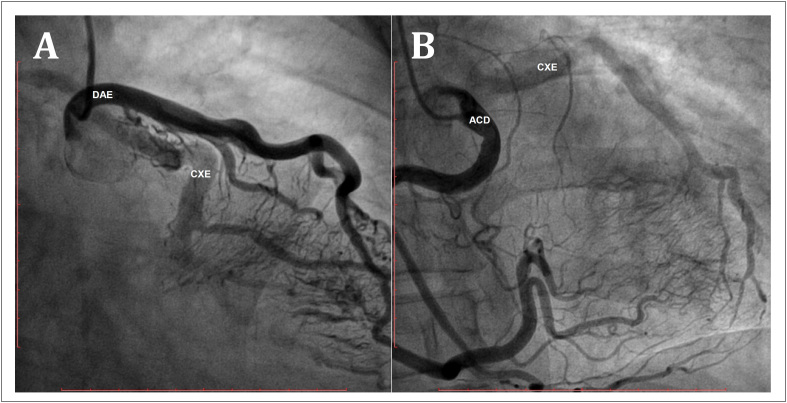
Angiografia coronária mostrando ectasia das artérias coronárias e uma rede extensa de colaterais originados na DAE (2A) e na ACD (2B) fornecendo perfusão retrógrada da CXE. ACD: artéria coronária direita; CXE: artéria coronária circunflexa esquerda; DAE: artéria descendente anterior esquerda.

A imagem da ATAC revelou artéria CXE anômala originando-se da AP proximal direita (Figura 1 A e B; seta). A Figura 1A ilustra uma imagem tridimensional renderizada por volume da árvore coronária, demonstrando a conexão anômala da CXE com a AP direita, cursando inferiormente à artéria coronária descendente anterior esquerda (DAE) proximal. Podemos observar também a origem normal da DAE e da artéria coronária direita (ACD), com dilatação das artérias principais, mas sem a observação de colaterais significantes. A Figura 1B é uma reconstrução multiplanar oblíqua com projeção de intensidade máxima de 1 mm de espessura, demonstrando a origem anômala da CXE de uma AP direita. Este achado foi confirmado por angiografia coronária subsequente, na qual foi observada ectasia das artérias coronárias, com uma rede extensa de colaterais originando-se da ACD e da DAE fornecendo perfusão retrógrada da CXE (Figura 2).

Nossa paciente apresentou angina de esforço, com teste de esforço positivo; portanto, ela foi encaminhada para cirurgia cardíaca. Foi realizada ligadura cirúrgica da artéria CXE anômala para diminuir o fluxo competitivo, o que pode causar falha do enxerto, seguida por enxerto de bypass com a artéria mamária interna esquerda para a CXE. Não houve complicações e a paciente permaneceu assintomática desde então.

## Discussão

A anatomia coronária normal é caracterizada por dois óstios localizados nos seios de Valsalva direito e esquerdo e é universalmente definida da maneira seguinte: a ACD origina-se do seio de Valsalva direito e a artéria coronária esquerda no seio esquerdo, geralmente abaixo da junção sinotubular e, com frequência, divide-se na artéria descendente anterior e na artéria circunflexa.^1^

Determinar o que é normal na anatomia das artérias coronárias constitui um desafio. Angelini et al.,^4^ classificaram qualquer anatomia presente em mais de 1% da população geral como normal. Assim, por definição, as anomalias congênitas das artérias coronárias (ACACs) ocorrem em menos de 1% da população.^3^ As ACACs foram descritas pela primeira vez há dois milênios por Galeno e Vesalius; são anormalidades na origem, estrutura e curso destas artérias.^3^

Existem diversas classificações. Clinicamente falando, as ACACs podem ser divididas em dois tipos, aquelas que causam instabilidade hemodinâmica significativa, ocorrendo em idade precoce e requerendo intervenção cirúrgica precoce, e aquelas que são assintomáticos até a velhice, que permanecem não identificadas, a menos que apresentem outros sintomas cardíacos ou sejam encontradas acidentalmente.^2^

A classificação inicialmente proposta por Angelini em 1989 tem sido posteriormente atualizada e é atualmente uma das mais utilizadas. Ela divide as ACACs em a) anomalias de origem e curso; b) anomalias da anatomia intrínseca das artérias coronárias; c) anomalias de terminação coronária; e d) vasos anastomóticos anômalos. O princípio básico desse sistema determina o nome de uma artéria pelo território para o qual ela fornece sangue e não baseado na sua origem ou curso inicial.^3^^,^^4^

A incidência real das ACACs na população geral permanece obscura; anomalias coronárias ocorrem em 0,3% a 0,9% dos pacientes sem doença cardíaca e em 3% a 36% daqueles com defeitos cardíacos estruturais.^1^ As ACACs são geralmente detectadas apenas na autópsia. Em atletas jovens, estas anomalias são a segunda causa mais comum de morte cardíaca súbita (em 12% de óbitos) e são geralmente desencadeados por exercícios físicos vigorosos.^3^

A síndrome de Bland-White-Garland ou a origem anômala da artéria coronária esquerda da artéria pulmonar (ALCAPA) foi descrita em 1933, após a autópsia de um neonato de 3 meses, com dificuldade de alimentação, cardiomegalia e evidência de lesão ventricular esquerda no ECG, sendo a anomalia coronária mais frequentemente associada à morte súbita.^1^^,^^3^

A ALCAPA tem uma incidência de 1 em 300.000 nascidos vivos. Esta anomalia é um importante diagnóstico diferencial em crianças com insuficiência cardíaca.^3^ Usualmente, apresenta-se como uma anomalia isolada mas, em 5% dos casos, pode estar associada a outras malformações (defeito septal, defeito septal ventricular ou coarctação da aorta) e, se não tratada, sua taxa de mortalidade no primeiro ano de vida é de 90%.^3^^,^^5^^,^^6^

De forma menos frequente, a ACD, a artéria coronária DAE ou a artéria coronária CXE têm sido relatadas como surgindo da AP em variantes mais raras desta síndrome.^7^^,^^8^

Os casos de origem anômala da artéria coronária direita da artéria pulmonar (ARCAPA) são extremamente raros, com uma incidência de 0,002%. Estas anormalidades são assintomáticas em mais de 75% de casos, sem evidência de isquemia miocárdica.^3^

A origem anômala da artéria CXE da artéria pulmonar (ALCxCAPA) pode ser considerada uma variante extremamente rara da ALCAPA, com o primeiro caso adulto relatado em 1992 por Garcia et al. e com pouco mais de 20 casos descritos na literatura até o momento presente.^7^^,^^9^ Está geralmente associada a outros defeitos cardíacos congênitos, com casos isolados sendo extremamente incomuns. Os casos descritos variam de neonatos a adultos, com apresentações clínicas variadas, incluindo relatos de sopro cardíaco assintomático, dispneia e angina. As formas mais graves encontradas na literatura incluem isquemia miocárdica, com poucos casos relatados de disfunção miocárdica grave e parada cardíaca secundária a esta anomalia.^1^^,^ ^2^

Durante o primeiro mês de vida, a hipertensão pulmonar fisiológica e a hemoglobina fetal fornecem perfusão e oxigenação ao miocárdio; consequentemente, estes indivíduos são assintomáticos.^3^ Em crianças maiores e adultos, as pressões relativamente baixas na artéria pulmonar normal criam um gradiente através do qual o sangue flui, direcionado da circulação coronária nativa, através da rede extensa de colaterais, para a artéria anômala e a artéria pulmonar. Isso resulta em fístula coronária-pulmonar, com o fenômeno do roubo coronário.^1^ Os pacientes tornam-se sintomáticos e podem apresentar angina, fadiga, dispneia, palpitações, arritmias ventriculares, hipertensão pulmonar e morte súbita.^3^ Os sintomas e o prognóstico dependem do desenvolvimento de vasos colaterais nas outras duas artérias.^1^^,^^2^

A nossa paciente permaneceu assintomática durante os primeiros 40 anos da sua vida. Nossa hipótese é que isso seja o resultado da combinação da área relativamente pequena do miocárdio fornecida pela artéria CXE, o grau de colateralização coronária e a falta de desafios cardíacos significativos anteriores.

A ATAC proporciona uma ferramenta de imagem não invasiva para demonstrar a origem e a relação das artérias anômalas com outras estruturas vasculares mediastinais e permite o uso de reformatação tridimensional para a delineação de variações sutis na posição e morfologia dos vasos anômalos. Além disso, desempenha um papel importante no planejamento de intervenção cirúrgica e pode ser uma ferramenta valiosa no acompanhamento pós-operatório para pacientes adultos.^10^

Foi demonstrado que a ATAC controlada por ECG foi superior à angiografia em termos de sensibilidade em várias séries.^6^ As Diretrizes de 2018 da ACC/AHA para o Manejo de Adultos com Doença Cardíaca Congênita recomendam o uso da ATAC como método de triagem para o diagnóstico e o manejo de pacientes com anomalias coronárias congênitas de origem arterial ectópica.^11^

A indicação de tratamento cirúrgico para origem anômala da CXE ainda não está bem estabelecida. Os critérios de tratamento são a presença de sintomas, a área ventricular que é suprida pela artéria e a colateralização a partir da DAE e/ou a ACD.^12^ Quando a cirurgia é indicada, a ligadura e o enxerto de bypass são recomendados em adultos; o reimplante promove resultados substancialmente melhores em bebês e crianças.^13^ No presente caso, considerando que a paciente apresentava angina e isquemia documentada, foi realizada a cirurgia.

## Conclusão

As ACACs constituem um grupo heterogêneo de anomalias congênitas raras, cujas manifestações variam muito. A origem anômala da artéria CXE da AP é ocultada pela presença de circulação colateral e pela área relativamente pequena suprida por este vaso. Embora a maioria dos pacientes com esta anomalia seja assintomática e seus exames físicos sejam normais, eles correm o risco de morte súbita. Consequentemente, esta condição requer um alto grau de suspeita clínica e a ATAC constitui-se como a modalidade de imagem de escolha.
