# Supplementary material for: Origem Anômala da Artéria Coronária Circunflexa da Artéria Pulmonar Direita: Diagnóstico por TC Cardíaca
Source: Arq Bras Cardiol. 2021 Jul 14;117(1 Supl 1):8–11. [Article in Portuguese] doi: 10.36660/abc.20200060 (PMC8291897; doi:10.36660/abc.20200060)
Supplement: Translation

# Translation: Anomalous Origin of the Circumflex Coronary Artery from the Right Pulmonary Artery: Diagnosis Through Cardiac CT

**Authors:** Bebiana Faria, Lucy Calvo, Sílvia Ribeiro, Catarina Ruivo, António Lourenço

**Affiliations:** 1 Guimarães Portugal Departamento de Cardiologia, Hospital da Senhora da Oliveira Guimarães, Guimarães - Portugal; 2 Leiria Portugal Departamento de Cardiologia, Leiria Hospital Center, Leiria - Portugal

**Keywords:** Coronary Artery Disease, Congenital Abnormalities, Heart Defects, Congenital, Coronary Vessels, Pulmonary Artery, Tomography, Computed/methods

## Introduction

Anomalous origin of the left circumflex coronary artery (LCx) from the right pulmonary artery (PA) is an extremely rare coronary anomaly. Although the clinical course may be silent, the risk of sudden cardiac death is increased. The symptoms are related to collateralization and amount of myocardium that it supplies, and some patients need surgical treatment.^1^^,^^2^

## Case report

A 44-year-old woman with a family history of Brugada syndrome and no other relevant medical background was examined in our hospital due to Canadian Cardiovascular Society class I exertional angina. She had no coronary risk factors and no family history of premature coronary artery disease or congenital heart disease.^3^ Physical examination was unremarkable. Transthoracic echocardiography only revealed mild mitral regurgitation, and ECG was normal.

She underwent an exercise echocardiogram that revealed wall motion abnormalities in the inferolateral wall, with worsening of the degree of mitral regurgitation. Brugada syndrome was excluded after pharmacological test with ajmaline.

Coronary computed tomography angiogram (CCTA) was performed (*Siemens Somatom Sensation 64 CT Scanner®*). A preliminary scan for scoring the amount of coronary calcium was obtained, and the Agatston score was zero. Seventy milliliters of iodinated contrast (*Ultravist 370®*) were administered. Nitroglycerin 0.3 mg was sublingually administered immediately before contrast injection. The patient was in sinus rhythm with a heart rate of 50 to 60 beats/min. A retrospective gated CCTA was performed, with reconstruction of cardiac phases at 70% of the R-R interval. The post-processing image was performed on *Aquarius Intuition TeraRecon®*.

CCTA imaging revealed an anomalous LCx arising from the proximal right PA (Figure 1 A and B; arrow). Figure 1A illustrates a three-dimensional volume-rendered image of the coronary tree demonstrating the anomalous connection of the LCx to the right PA, coursing inferior to the proximal left anterior descending (LAD) coronary artery. We can also observe the normal origin of the LAD and right coronary artery (RCA), with dilation of the main arteries, but no significant collaterals are observed. Figure 1B is an oblique multiplanar reconstruction with maximum intensity projection 1 mm thick, demonstrating the anomalous origin of the LCx from a right PA. This finding was confirmed on a subsequent coronary angiogram, where ectasia of the coronary arteries was observed, with an extensive network of collaterals originating in the RCA and LAD supplying retrograde perfusion of the LCx (Figure 2).

**Figure 1:** Multislice cardiac CT using a 64-slice scanner showing anomalous origin of the circumflex coronary artery from the right pulmonary artery (arrow). LCx: left circumflex coronary artery; LAD: left anterior descending artery; RPA: right pulmonary artery; Ao: aorta; RCA: right coronary artery; OM1: first obtuse marginal.

**Figure 2:** Coronary angiogram showing ectasia of the coronary arteries and an extensive network of collaterals originating in the LAD (2A) and RCA (2B) supplying retrograde perfusion of the LCx. LCx: left circumflex coronary artery; LAD: left anterior descending artery; RCA: right coronary artery.

Our patient had exertional angina, with a positive stress test; therefore, she was referred for cardiac surgery. Surgical ligation of the anomalous LCx was performed to decrease competitive flow, which can cause the graft to fail, followed by coronary bypass graft with left internal mammary artery to the LCx. There were no complications and the patient remained asymptomatic since then.

## Discussion

Normal coronary anatomy is characterized by two ostia located in the right and left Valsalva sinuses and is universally defined as follows: the RCA originates from the right Valsalva sinus and the left coronary artery in the left sinus, usually below the sinotubular junction, and it usually divides into the anterior descending artery and the circumflex artery.^1^

Determining what is normal in the anatomy of coronary arteries is a challenge. Angelini et al.^4^ classified any anatomy present in more than 1% of the general population as normal. Thus, by definition, congenital coronary artery anomalies (CCAAs) occur in less than 1% of the population.^3^ CCAAs were first described two millennia ago by Galen and Vesalius and are abnormalities in the origin, structure, and course of these arteries.^3^

There are several classifications. Clinically speaking, CCAAs can be divided into two types, those that cause significant hemodynamic instability, occurring at an early age and requiring early surgical intervention, and those that are asymptomatic until old age, which remain unidentified unless they present with other cardiac symptoms or are found accidentally.^2^

The classification initially proposed by Angelini in 1989 has subsequently been updated, and it is currently one of the most used. It divides CCAAs into a) anomalies of origination and course; b) anomalies of intrinsic coronary arterial anatomy; c) anomalies of coronary termination; and d) anomalous anastomotic vessels. The basic principle of this system is that the name of an artery is determined by the territory to which it supplies blood and not based on its origin or initial course.^3^^,^^4^

The real incidence of CCAAs in the general population remains unclear; coronary anomalies occur in 0.3% to 0.9% of patients without heart disease and in 3% to 36% of those with structural heart defects.^1^ CCAAs are often only detected in autopsy. In young athletes, these anomalies are the second most common cause of sudden cardiac death (in 12% of deaths), and they are generally triggered by vigorous physical exercise.^3^

Bland-White-Garland syndrome or anomalous origin of the left coronary artery from the pulmonary artery (ALCAPA) was described in 1933, following the autopsy of a 3-month-old infant with difficulty feeding, cardiomegaly and evidence of left ventricular lesion on ECG, and it is the coronary anomaly most often associated with sudden death.^1^^,^^3^

ALCAPA has an incidence of 1 in 300,000 live births. This anomaly is an important differential diagnosis in children with heart failure.^3^ It usually presents as an isolated anomaly, but, in 5% of cases, it may be associated with other malformations (septal defect, ventricular septal defect, or coarctation of the aorta), and, if left untreated, its mortality rate in the first year of life is 90%.^3^^,^^5^^,^^6^

Less commonly, the RCA, LAD coronary artery, or LCx coronary artery have been reported to arise from the PA in rarer variants of this syndrome.^7^^,^^8^

Anomalies in the origin of the right coronary artery from the pulmonary artery (ARCAPA) are extremely rare, with an incidence of 0.002%. These abnormalities are asymptomatic in more than 75% of cases, with no evidence of myocardial ischemia.^3^

Anomalous origin of the LCx artery from the pulmonary artery (ALCxCAPA) can be considered an exceedingly rare variant of ALCAPA, with the first adult case reported in 1992 by Garcia et al. and with just over 20 cases described in the literature to date.^7^^,^^9^ It is usually associated with other congenital heart defects, with isolated cases being extremely uncommon. The described cases range from neonates to adults, with varied clinical presentations, including reports of asymptomatic heart murmur, dyspnea, and angina. The most severe forms found in the literature include myocardial ischemia, with few reported cases of severe myocardial dysfunction and cardiac arrest secondary to this anomaly.^1^^,^^2^

During the first month of life, physiological pulmonary hypertension and fetal hemoglobin provide perfusion and oxygenation to the myocardium; consequently, individuals are asymptomatic.^3^ In older children and adults, relatively low pressures in the normal pulmonary artery create a gradient through which blood flows, directed from native coronary circulation, through the extensive collateral network, to the anomalous artery and pulmonary artery. This results in coronary-pulmonary artery fistula, with the coronary steal phenomenon.^1^ Patients become symptomatic and may experience angina, fatigue, dyspnea, palpitations, ventricular arrhythmias, pulmonary hypertension, and sudden death.^3^ Symptoms and prognosis depend on the development of collateral vessels in the other two arteries.^1^^,^^2^

Our patient remained asymptomatic throughout the first 40 years of her life. We hypothesize that this is a result of the combination of the relatively small area of myocardium supplied by the LCx artery, the degree of coronary collateralization, and the lack of significant previous cardiac challenges.

CCTA provides a noninvasive imaging tool to demonstrate the origin and relationship of anomalous arteries to other mediastinal vascular structures, and it enables the use of three-dimensional reformation for delineation of subtle variations in the position and morphology of anomalous vessels. Moreover, it plays an important role in surgical intervention planning, and it may be a valuable postoperative follow-up tool for adult patients.^10^

ECG-gated CCTA was shown to be superior in sensitivity to invasive angiography in several series.^6^ The ACC/AHA 2018 Guidelines for the Management of Adults with Congenital Heart Disease recommend the use of CCTA as a screening method for diagnosis and patient management in congenital coronary anomalies of ectopic arterial origin.^11^

The indication for surgical treatment of anomalous origin of the LCx is not yet well established. The criteria for treatment are the presence of symptoms, the ventricular area that is supplied by the artery, and collateralization from the LAD and/or the RCA.^12^ When surgery is indicated, ligation and bypass grafting are recommended in adults; re-implantation yields substantially better results in infants and children.^13^ In this case, as the patient had angina and documented ischemia, surgery was performed.

## Conclusion

CCAAs are a heterogeneous group of rare congenital abnormalities, whose manifestations vary greatly. The anomalous origin of the LCx artery from the PA is masked by the presence of collateral circulation and the relatively small area supplied by this vessel. Although most of the patients with this anomaly are asymptomatic and their physical examinations are unremarkable, they are at risk of sudden death. As such, this condition requires a high degree of clinical suspicion and CCTA is the imaging modality of choice.
